# One-Step Synthesis of Metastable Mo_2_AlB_2_ from MoAlB Using Gaseous HCl

**DOI:** 10.1021/acs.inorgchem.4c04794

**Published:** 2025-01-06

**Authors:** Tugser Yilmaz, Ozden Gunes Yildiz, Naeimeh Sadat Peighambardoust, Michael Baitinger, Umut Aydemir

**Affiliations:** a Graduate School of Sciences and Engineering, Koç University, Istanbul 34450, Türkiye; b Koç University Boron and Advanced Materials Application and Research Center, Istanbul 34450, Türkiye; c Max-Planck Institute for Chemical Physics of Solids, Nöthnitzer Strasse 40, Dresden 01187, Germany; d Department of Chemistry, Koç University, Sariyer, Istanbul 34450, Türkiye

## Abstract

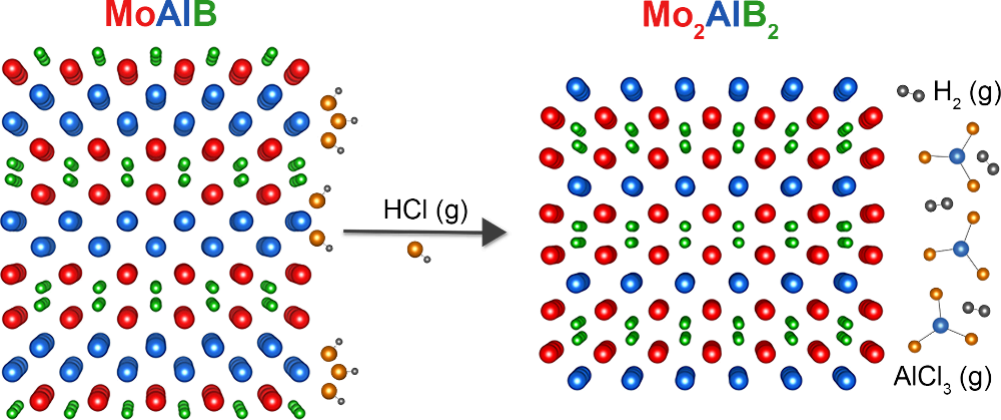

Recent studies increasingly highlight the potential applications of MBenes, a novel class of two-dimensional (2D) materials, yet their production remains challenging. In this context, microcrystalline Mo_2_AlB_2_ (space group *Cmmm*; *a* = 3.080(3) Å, *b* = 11.57(1) Å, and *c* = 3.149(4) Å), a promising precursor for MoB MBene production, was synthesized in a single-step gas–solid reaction at 450 °C using MoAlB and gaseous HCl. This preparation method, previously utilized for the oxidation of Zintl phases, has been successfully adapted to compounds containing *d*-block elements, providing a new alternative for exfoliating layered materials in liquid solutions. Scanning electron microscopy analysis revealed homogeneous products with microcrystals exhibiting a nonuniform particle size distribution. At higher temperatures, these evolved into plate-like crystallites with smooth surfaces and etch cavities. This efficient and cost-effective gas–solid reaction shows great potential for large-scale production of a wide range of 2D materials, with significant benefits for catalysis, energy storage, and other applications.

## Abstract 2

This study introduces a scalable, one-step gas−solid reaction method for synthesizing metastable Mo_2_AlB_2_ from MoAlB using gaseous HCl. The process operates at a moderate temperature of 450 °C and effectively removes aluminum as volatile AlCl_3_. This innovative approach offers a versatile and efficient route for producing 2D materials like MBenes, paving the way for advancements in catalysis, energy storage, and beyond.

## 1 Introduction

2D materials can be synthesized from various sources, including van der Waals (vdW) materials such as graphite,^1^ black phosphorus,^2^ and metal chalcogenides^3^ as well as from hybrid bonded (including covalent, ionic, and metallic bonding) structures like MAX phases.^4^ Exfoliation of such materials is possible due to the lower energy requirements associated with their weak interlayer interactions. In contrast, extracting 2D materials from hybrid bonded three-dimensional (3D) materials poses a significant challenge, as it requires overcoming significantly higher energy barriers.^5^ Among these materials, the exfoliation of MAX phases has been explored to produce 2D MXenes, which have garnered extensive interest and study over recent years.^6^ In MAX compounds, the acronym “MAX” reflects their composition: *M* stands for an early transition metal, *A* for an element from group 13 or 14, and *X* for carbon or nitrogen. In MAX compounds, the crystal structures are composed of covalently bonded *MX* layers, connected by *MA* layers with more metallic bonding character. In various cases, selective etching of *A* atoms has been accomplished, resulting in 2D nanosheets called MXenes (formula *MXT*_*x*_), terminated with surface groups *T*_*x*_.^7,8^

**Scheme 1:**
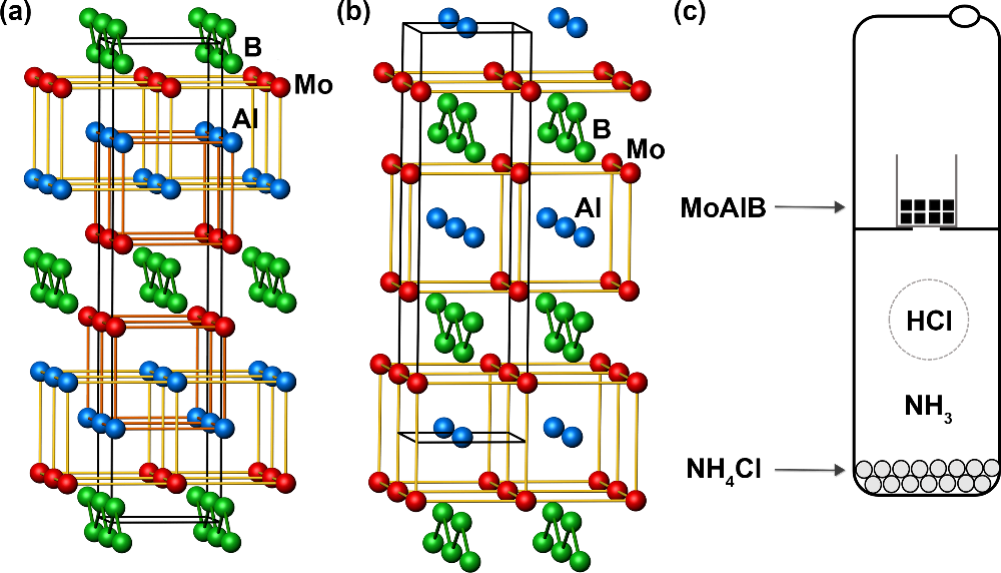
Crystal Structures of (a) MoAlB (*Cmcm*)^17^ and (b) Mo_2_AlB_2_ (*Cmmm*); (c) Schematic Representation of the Reactor

Recently, boron-based analogs of MXenes, known as MBenes, have attracted research interest due to their potential applications in, e.g., catalysis and battery technology.^9−13^ In these compounds, boron atoms replace the *X* atoms and are synthesized from layered ternary transition metal borides, referred to as MAB phases.^14,15^ MAB phases typically exhibit orthorhombic or hexagonal crystal structures, where *M-B* blocks are separated by single or bilayer *A* elements.^16^ As a member of the MAB phases, MoAlB is the only thermodynamically stable phase in the ternary system Mo–Al–B.^17^ In its orthorhombic crystal structure, Mo–B layers are separated from each other by a double layer of Al (Scheme 1a). In addition, a metastable compound, Mo_2_AlB_2_,^9^ has been obtained with a closely related orthorhombic crystal structure in which only a single Al layer separates the MoB layers (Scheme 1b). This single-layer configuration makes Mo_2_AlB_2_ a more suitable precursor for synthesizing 2D MoB^18^ (MBene) compared to MoAlB. Therefore, identifying scalable synthetic routes for the metastable Mo_2_AlB_2_ phase would be advantageous.

Various methods have been explored to deintercalate Al in MoAlB to obtain Mo_2_AlB_2_ (Table 1). Aqueous solutions of HF and LiF + HCl, although effective at relatively low temperatures, require extended reaction times of up to 48 h, along with additional drying and phase separation steps and involve hazardous chemicals, raising significant health and safety concerns.^19,20^ NaOH-based methods require an additional heat treatment step at 600 °C for 4.5 h, increasing both process complexity and duration.^21^ Lewis acid molten salt techniques, like those involving ZnCl_2_,^22^ require high temperatures and post-treatment with aqueous HCl to remove residual Zn nanoparticles. Similarly, CuCl_2_-based methods demand even higher temperatures and further washing steps with ammonium persulfate ((NH_4_)_2_S_2_O_8_).^23^ These methods often involve prolonged reaction times, high temperatures, or extensive postprocessing, highlighting the need for a more efficient, single-step synthesis route.

**Table 1:** Comparative Summary of Synthesis Methods for Mo_2_AlB_2_, Showing Experimental Techniques and Conditions

**reaction medium**	**temperature (°C)**	**duration (h)**	**additional treatment**	**reference**
gaseous HCl	450	2	-	this work
HF solution	45	48	-	(19)
LiF + HCl solution	40	48	-	(20)
NaOH solution	Room temperature	24	Heat treatment	(21)
ZnCl_2_ molten salt	550	2	HCl treatment	(22)
ZnCl_2_ molten salt	550	170	-	(24)
CuCl_2_ molten salt	650	2	(NH_4_)_2_S_2_O_8_ treatment	(23)

In our previous studies, we explored converting reactive Zintl phases into metastable or hard-to-access stable phases using gaseous oxidizing agents at moderate reaction temperatures.^25,26^ For instance, as an alternative to the well-established high-pressure synthesis of the clathrate-I phase Ba_8–*x*_Si_46_, the precursor Ba_4_Li_2_Si_6_ was oxidized at low temperature and ambient pressure by reaction with gaseous HCl. The coproducts BaCl_2_ and LiCl were washed from the product.^27^ However, this method has not yet been applied to stable compounds containing *d*-block elements. Applying this method to convert MoAlB to Mo_2_AlB_2_ may offer a significant advantage, as the coproduct AlCl_3_ evaporates, eliminating the need for washing and preventing solvent contamination on the crystallite surfaces.

In this study, we present the conversion of MoAlB to the metastable Mo_2_AlB_2_ phase through a topochemical synthesis using gaseous HCl in a closed system (Scheme 1c). This method offers a promising gas–solid reaction pathway that may enable the direct synthesis of 2D MBene materials from other MAB phases. By addressing key challenges in the stabilization and exfoliation of hybrid bonded structures, this approach offers a scalable and efficient route for developing MBenes, opening avenues for their broad application in fields such as catalysis and energy storage.

## 2 Experimental Section

### 2.1 Characterization

The structure and phase analysis were carried out by a Rigaku Mini Flex 600 X-ray diffractometer (XRD) equipped with a Cu Kα radiation (λ = 1.5418 Å). XRD patterns were obtained within the 2θ range of 10–90°, employing a scanning rate of 5° s^–1^. The morphology was investigated via field emission-scanning electron microscopy (FE-SEM, Zeiss Ultra Plus) connected to an energy-dispersive X-ray spectroscopy (EDX) detector (Bruker XFlash 5010, 123 eV spectral resolution). High-resolution transmission electron microscopy (HR-TEM) images were captured using a Thermo Scientific Talos F200S TEM 200 kV instrument. To gain better insights into the surface composition and oxidation states, X-ray photoelectron spectroscopy (XPS) was conducted using a Thermo Scientific K-Alpha instrument equipped with an Al Kα monochromator source emitting at 1486.6 eV. All XPS spectra underwent correction based on the binding energy of C 1s, set at 284.50 eV. The chemical composition of the prepared powder was analyzed using an Agilent 7700x Inductively Coupled Plasma-Mass Spectrometry (ICP-MS) system.

### 2.2 Preparation

#### α-MoB

A polycrystalline powder specimen of α-MoB was synthesized via carbothermal reduction.^28−30^ MoO_3_ (powder, 99.9% metals basis, Alfa Aesar), B_2_O_3_ (powder, 99.9% metals basis, Alfa Aesar), and activated charcoal (powder, pure, Sigma-Aldrich) were weighed in stoichiometric amounts under an Ar atmosphere in a glovebox. This mixture was loaded into a hardened stainless-steel vial and sealed. The steel vials were cleaned before by using silica in a sandblaster. High-energy ball milling was employed for 3 h using steel balls of two 1/2″ and four 1/4″ in diameter, with a ball-to-powder weight ratio of 13:1. After milling, the mixture was observed to be homogeneous. The mechanically activated powder was transferred to a graphite crucible and heated at a rate of 8 °C/min to 1450 °C and annealed for 6 h at this temperature. Heat treatment was performed in a horizontal tube furnace under flowing Ar. The resulting product formed according to eq 1 was single phase α-MoB according to powder X-ray diffraction (PXRD) (Figure S1). The crystallinity of the product was also confirmed by scanning electron microscope (SEM) images (Figure S2).
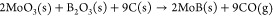
1

#### MoAlB

MoB and Al (powder, 99.5% metals basis, Alfa Aesar) were weighed in a 1:1.6 ratio under an Ar atmosphere in glovebox and transferred in a hardened stainless-steel vial. To homogenize the powder mixture, high-energy ball milling was performed for 5 min using two 1/4″ diameter steel balls with a ball-to-powder ratio of 3:1. Subsequently, the mixture was pressed into a pellet with a diameter of 10 mm by applying 5 tons of force. The pellet was placed in an alumina crucible and heated in a horizontal tube furnace at a rate of 8 °C/min to 1200 °C and annealed for 1 h at this temperature under constant Ar flow. After the reaction, the pellets were removed from the furnace and ground into powder using a tungsten carbide mortar for 30 min. The resulting product was found to be single phase according to PXRD (Figure S3). SEM images of the MoAlB product show microcrystals with nonuniform particle size distribution (Figure S4).

#### Mo_2_AlB_2_

To prepare Mo_2_AlB_2_ using HCl solutions, 0.2 g of MoAlB powder was initially mixed with 50 mL of HCl at varying concentrations ranging from 1 to 3 M for durations of 1 to 6 h. The mixture was then centrifuged to separate the solid phase, and the resulting powder was washed with deionized (DI) water until the solution reached a pH of 7. Finally, the powder was washed with ethanol and dried in a vacuum oven at 60 °C for 12 h.

**Figure 1:**
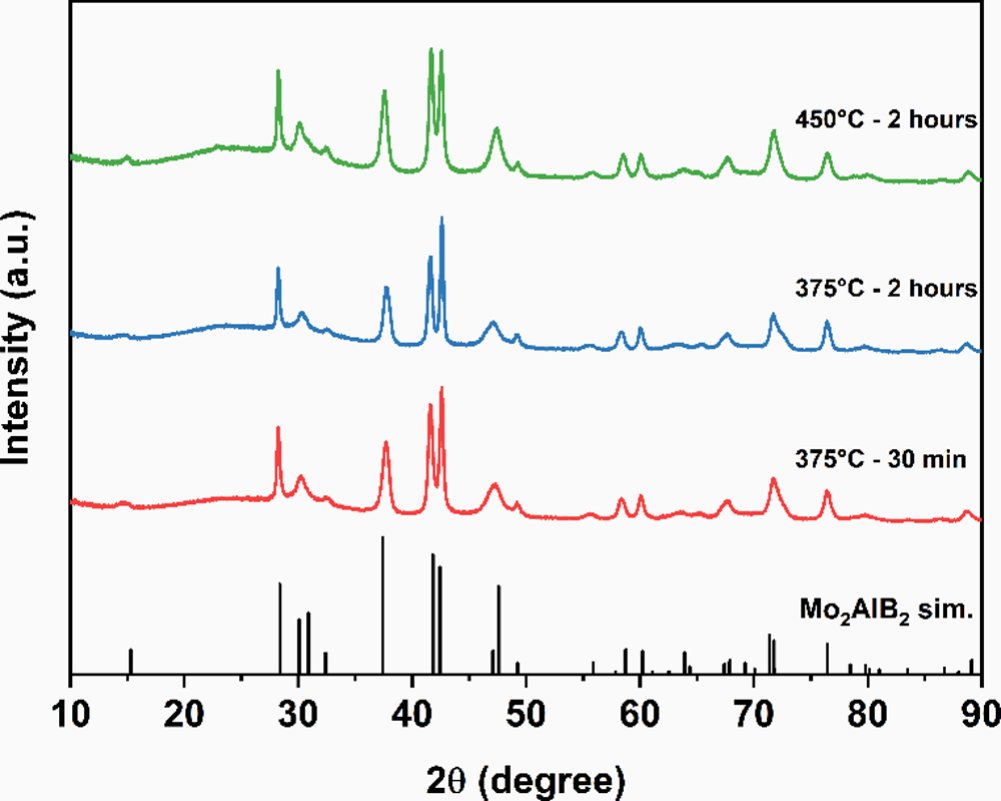
Mo_2_AlB_2_ products obtained from the reaction of MoAlB and gaseous HCl at different annealing conditions.

For the synthesis with gaseous HCl, all steps aside from sealing and washing were conducted under a protective Ar atmosphere in a glovebox. For this process, 1 mmol of MoAlB and 2 mmol of NH_4_Cl (99.5%, Sigma-Aldrich) were weighed and MoAlB was placed in an 8 mL glass vial. Prior to use, NH_4_Cl was dried at 150 °C for 2 h in a Pyrex glass crucible under vacuum. The vial was then positioned in a glass reactor, which was promptly sealed under vacuum together with NH_4_Cl (Scheme 1c and Figure S5). The sealed reactor was inserted into a steel tube, insulated with glass wool at both ends, and positioned in a vertical furnace. Heat treatment was applied at temperatures between 375 and 450 °C (heating rate: 3 °C/min) for 30 to 120 min. After heating, the samples were slowly cooled by turning off the furnace. Upon reaching room temperature, the reactor was opened, and the samples were washed with DI water and ethanol, then dried in a vacuum oven at 60 °C for 12 h. PXRD confirmed the products to be single-phase (Figure 1).

## 3 Results and Discussion

To examine the impact of aqueous HCl on MoAlB, etching experiments were conducted using 1 and 3 M HCl solutions at various temperatures and durations, focusing on their effects on the phase formation (Figure S6). Initial trials with 1 M HCl revealed significant alterations in the XRD patterns, pointing to notable structural changes. Extended tests with both 1 and 3 M HCl for 6 h further explored temperature effects on these modifications. The (0*k*0) reflections, related to the stacking of MoB and Al layers, displayed shifts, broadening, or complete disappearance,^31^ indicating disruptions in the long-range order along the [0*k*0] crystallographic direction, likely due to stacking faults caused by the etching process.^21^ These observations imply reduced crystalline coherence, increased defect formation, and stacking disorder, underscoring the impact of HCl etching on structural integrity.

Further insights were gained by comparing peak positions, particularly for the (020) plane, as highlighted in Figure S6. In tests conducted with 1 M HCl for 1 h, no significant changes were observed apart from peak broadening. However, extending the duration to 6 h caused a peak shift for the (020) plane to approximately 13°, alongside amorphization, likely corresponding to intermediate MoAl_1–*x*_B phases, such as Mo_4_Al_3_B_4_, identified by Kim et al.^20^ and supported by Alameda et al.,^18^ who noted the formation of phases like Mo_6_Al_5_B_6_, Mo_4_Al_3_B_4_, and Mo_3_Al_2_B_3_ during chemical etching. Notably, neither higher temperatures nor increased HCl molarity significantly affected the diffraction pattern in the liquid–solid reactions. To investigate the resulting composition after 6 h HCl treatment, SEM-EDX analysis was conducted (Table S1), revealing an approximate atomic ratio of *n*(Mo):*n*(Al) = 1.5, indicating that the transformation from MoAlB to Mo_2_AlB_2_ was not complete.

Due to the limited success of the solution-phase HCl etching experiments, we shifted to using gaseous HCl to investigate its efficiency in facilitating the transformation. Thus, the precursor phase MoAlB and NH_4_Cl were reacted at temperatures ranging from 375 to 450 °C. At these temperatures, NH_4_Cl is completely decomposed into HCl and NH_3_.^32^ Since the reactants are spatially separated within the reactor (Scheme 1c), the reaction solely occurs via the gas phase, thus constituting a heterogeneous gas–solid reaction. Primarily, HCl reacts with MoAlB (eq 2):
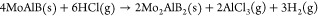
2

The reaction temperatures required for the transformation of MoAlB are surprisingly low: higher temperatures were required for the oxidation of the reactive Zintl phase Ba_4_Li_2_Si_6_ to Ba_8–*x*_Si_46_ applying the same method.^27^ An important driving force for the reaction of MoAlB with HCl is likely the formation of gaseous AlCl_3_. Gaseous NH_3_, which is the other component in the gas phase, may act as a protic oxidizing agent as well.^33^

For all reaction temperatures, the crystalline reaction product only consisted of Mo_2_AlB_2_, according to PXRD (Figure 1). The comparative analysis of the gaseous HCl experiments with those utilizing HCl solutions reveals significant alterations in reflection positions, alongside the emergence of new peaks, indicating a distinct impact of the gaseous environment on the structural transformations of MoAlB (Figure S6). All reflections were indexed using the structure model obtained by Zhou et al. through quantum chemical optimization.^34^ The pronounced reflection broadening observed in the diffraction patterns is expected for a low-temperature conversion, which typically results in low crystallinity. In addition, a complex arrangement of crystalline domains can cause dramatic line broadening. In this respect, the characteristics of the diffraction patterns are reminiscent to the highly complex γ-Al_2_O_3_ structure.^35−37^ A simple Rietveld refinement did not allow us to obtain a reliable structure model. Lattice parameters were determined approximately based on 25 reflections (Table 2 and Table S2). The refinement revealed that reflections of lattice planes nearly perpendicular to the stacking axis show the largest deviation in 2θ. Therefore, it is possible that in the real microstructure, some of the Al double layers are still present, separating ideal crystalline domains above and below, which mostly affects the *b* cell parameter. Resolving this issue requires detailed structural investigations, which were beyond the scope of this work.

**Table 2:** Lattice Parameters of Mo_2_AlB_2_ (Space Group *Cmmm*) in Comparison with Literature Values

*a***(Å)**	*b***(Å)**	*c***(Å)**	**reference**
3.080(3)	11.57(1)	3.149(4)	this work
3.0610(2)	11.4200(8)	3.1428(1)	exp^38^
3.07	11.5	3.18	exp^21^
3.079	11.520	3.144	exp^22^
3.06	11.5	3.18	sim^19^
3.0710	11.5706	3.1413	sim^34^
3.0747	11.4619	3.1703	sim^22^
3.078	11.551	3.148	sim^9^
3.08914	11.63432	3.12544	sim^39^

**Figure 2:**
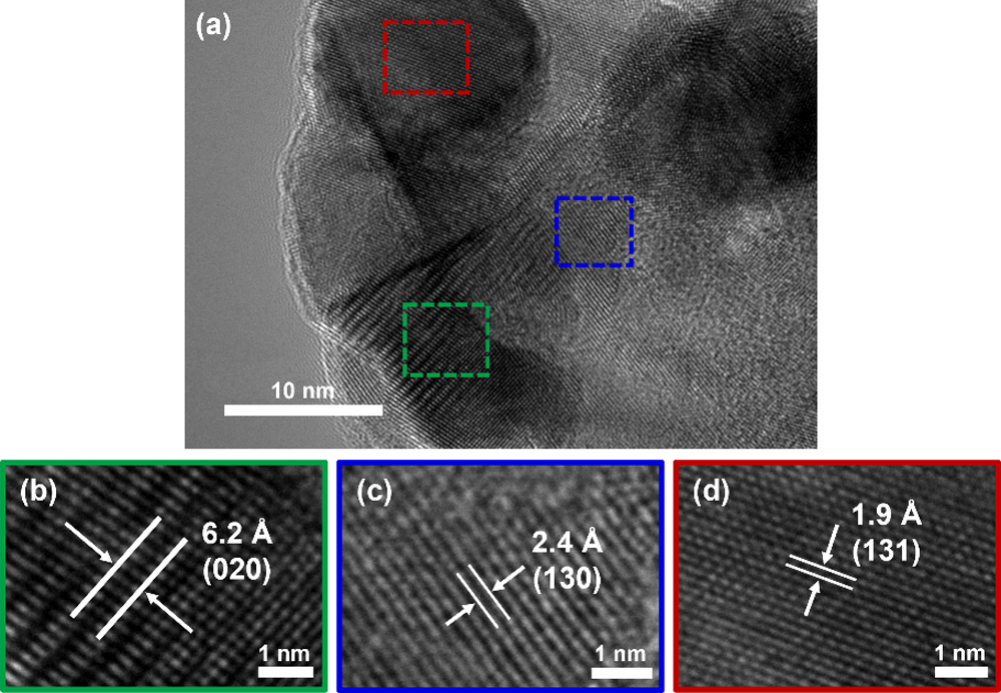
HR-TEM images of (a) Mo_2_AlB_2_ with different *d* spacings corresponding to (b) (020), (c) (130), and (d) (131) planes.

The lattice spacing for Mo_2_AlB_2_ was further investigated by HR-TEM, indicating that the structure model originally proposed by Guo et al. is valid,^9^ at least within separate crystalline domains (Figure 2a). The interlayer spacing corresponding to the (020) plane is reduced from 7.2 Å in MoAlB to 6.2 Å in Mo_2_AlB_2_ (Figure 2b and Figure S7). These results are consistent with the XRD findings (Figure S3 and Table S2), which show the 2θ angle of the (020) plane shifting from 12.66° to 14.99°, indicating a decrease in the interlayer distance from 6.99 to 5.91 Å. Additionally, the HR-TEM image of Mo_2_AlB_2_ reveals *d*-spacings of 2.4 Å (2.41 Å in XRD) and 1.9 Å (1.91 Å in XRD) corresponding to the (130) and (131) planes, respectively (Figure 2c,d).

**Figure 3:**
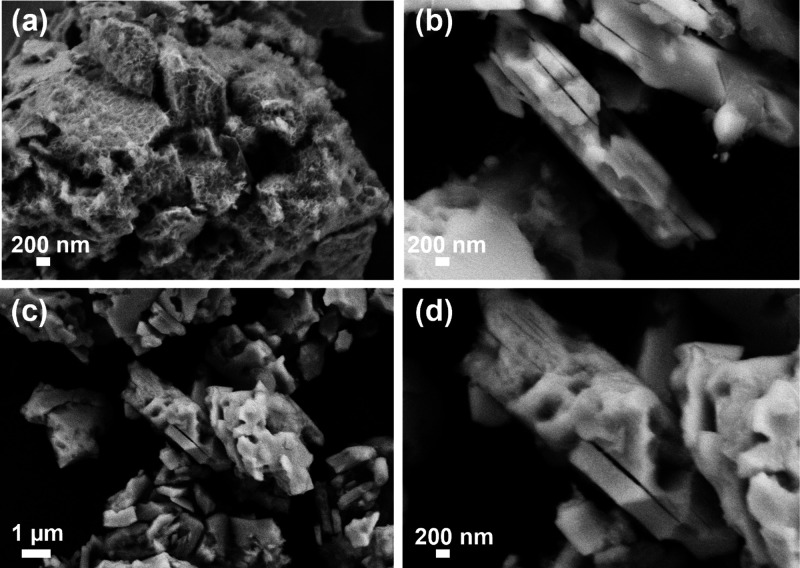
SEM images of Mo_2_AlB_2_ treated at 375 °C for (a) 30 min and (b) 2 h and at 450 °C for 2 h at (c) lower magnification and (d) higher magnification.

SEM images (Figure 3) show the homogeneity of the product, confirming that the conversion from MoAlB to Mo_2_AlB_2_ can be favorably performed in a one-step gas–solid reaction. After reaction at 375 °C for 30 min (Figure 3a), the particle surface appears highly porous. When the heat treatment was extended for 2 h (Figure 3b), the products consisted of small, plate-like crystallites with smooth surfaces containing etch cavities. Heat treatment at 450 °C for 2 h revealed similar characteristics but improved crystallinity (Figure 3c,d).

**Figure 4:**
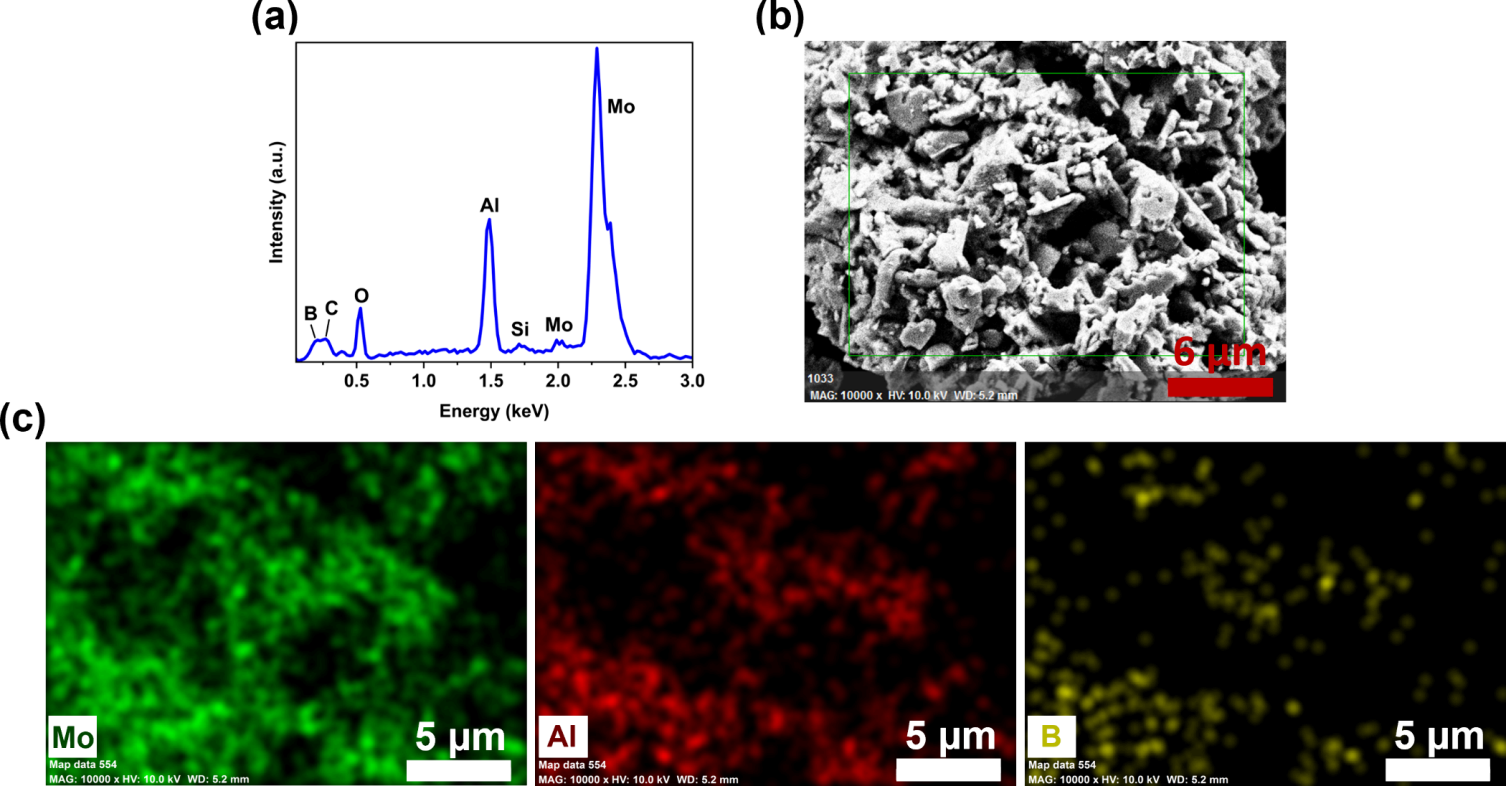
SEM-EDX investigation of Mo_2_AlB_2_. (a) Image with SE contrast; (b) EDX spectrum; (c) elemental mapping of Mo, Al, and B.

EDX revealed the expected signals of Mo, Al, and B, but also of Si, C and O (Figure 4a). The Si signal is attributed to impurities from the glass ampule or residues of silica sand used to clean the stainless-steel vials, while the C signal stems from the carbon tape of the sample holder and O to surface oxidation. The distribution of the majority components Mo, Al, and B in the sample is homogeneous (Figure 4b,c). A quantitative analysis of light elements such as boron is not reliable by EDX, moreover the arbitrarily oriented grains only allow for an estimation, but the determined atomic ratio of *n*(Mo):*n*(Al) ≈ 2.5 roughly corresponds to Mo_2_AlB_2_ (Table S3). From ICP-MS analysis the composition of the product was determined to be *n*(Mo):*n*(Al):*n*(B) = 2:0.8:1.8.

**Figure 5:**
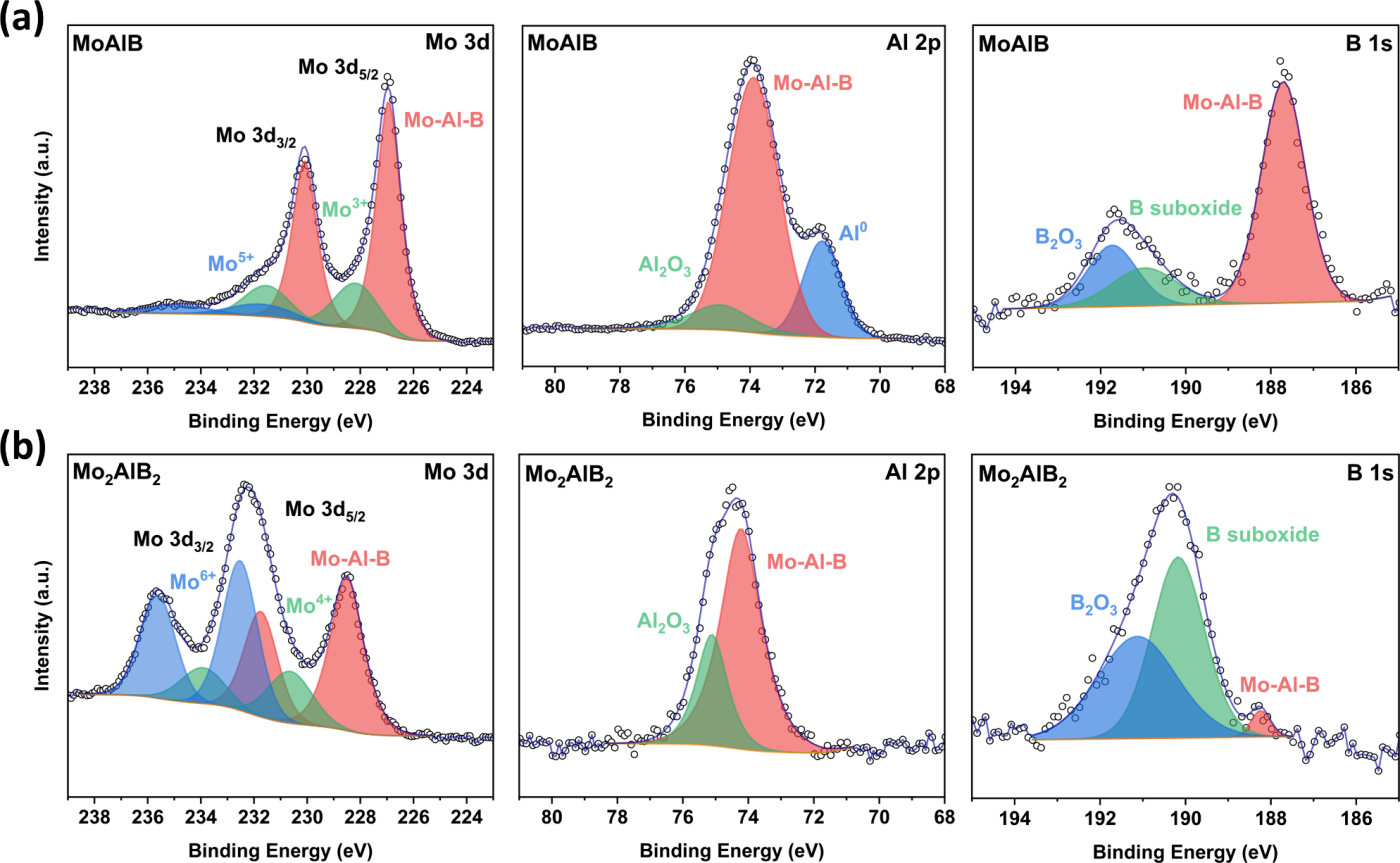
High-resolution XPS spectrum for Mo, Al, and B elements of (a) MoAlB and (b) Mo_2_AlB_2_.

X-ray photoelectron spectroscopy (XPS) for MoAlB and Mo_2_AlB_2_ supports the EDX results (Figure 5). The spectra were fitted to Mo 3d, Al 2p, and B 1s states. In the Mo 3d spectrum of MoAlB, three sets of doublets are observed at 226.94, 228.18, and 231.74 eV, corresponding to the Mo–Al–B, Mo^3+^, and Mo^5+^ states. For Mo_2_AlB_2_, the peaks are located at 228.51, 230.64, and 232.53 eV and are attributed to the Mo–Al–B, Mo^4+^, and Mo^6+^, respectively.^40^ The shift to higher binding energy by nearly 1.5 eV for MoAlB and the increase in oxidation states of Mo might be explained by the lower Al content, which causes a change in the valence state of Mo. The Al 2p spectra of MoAlB showed three individual peaks assigned to elemental Al, MoAlB, and Al_2_O_3_ at binding energies of 71.78, 73.89, and 74.92 eV, respectively.^41,42^ Metallic Al is observed because excess Al is used in the synthesis of MoAlB to compensate for the loss of Al during synthesis due to high-temperature volatilization.^43,44^ The Al 2p signals for Mo_2_AlB_2_ were deconvoluted at 74.22, and 75.11 eV, corresponding to Mo_2_AlB_2_, and Al_2_O_3_. The decrease in the peak area in the high-resolution spectra of Al 2p of Mo_2_AlB_2_ compared to MoAlB (Figure 5 and Figure S8) can be considered evidence of partial deintercalation of Al. The fitted curve of the B 1s states for MoAlB and Mo_2_AlB_2_ resulted in binding energies of 187.7 and 188.17 eV, respectively. Additionally, signals corresponding to boron suboxide (B_6_O) and B_2_O_3_ are detected in both MoAlB and Mo_2_AlB_2_. The binding energies associated with boron suboxide are observed at around 190 eV, while the binding energies corresponding to B_2_O_3_ were identified at 191.73 eV for MoAlB and 191.13 eV for Mo_2_AlB_2_.^45^

The XPS spectra for both MoAlB and Mo_2_AlB_2_ reveal multiple oxidation states of molybdenum, along with the presence of aluminum and boron oxides, suggesting significant surface oxidation of the fine powders exposed to air after synthesis.^46^ The washing process with deionized water and ethanol may have further contributed to this surface oxidation. Washing was performed before the XPS experiments to make sure that a possible precipitation of AlCl_3_ in the closed reactor does not contaminate the sample surface. Notably, PXRD analysis didn't show crystalline oxide or AlCl_3_ peaks, indicating that the oxidation is primarily limited to the surface and does not significantly impact the bulk phase. To mitigate these effects, conducting the reaction in flowing HCl would eliminate the need for washing and potentially reduce surface oxidation.

The approach introduced herein is particularly relevant for the transformation from MoAlB to Mo_2_AlB_2_, wherein a critical step involves breaking the Al–Al bond by removing a single layer of aluminum using gaseous HCl. Given the structural similarity between e.g., WAlB and MoAlB, both classified as *M*_2_Al_2_B_2_ type MAB phases,^14^ aluminum deintercalation could also be feasible for WAlB, suggesting the possibility of a similar transformation. Bond strength comparisons reveal that the energy required to break the Al–Al bond falls within the capabilities of gaseous HCl,^9^ facilitating the transformation from *M*_2_Al_2_B_2_ to *M*_2_AlB_2_.

## 4 Conclusions

In this study, we successfully applied a one-step gas–solid reaction to synthesize the metastable MAB phase Mo_2_AlB_2_, a method previously used for reactive Zintl phases and now adapted for d-block element compounds. This approach, using NH_4_Cl to generate gaseous HCl in situ, enables a straightforward and scalable synthesis route, efficiently removing aluminum as AlCl_3_ without requiring additional drying or separation. Compared to other methods such as aqueous HF, HCl, and NaOH solutions or high-temperature molten salt approaches, this gaseous HCl method operates at lower temperatures, reduces process complexity, and overcomes key limitations of existing techniques. The versatility and efficiency of this gas–solid synthesis pave the way for broader applications in, e.g., catalysis, energy storage, and beyond. As a simple and adaptable process, it serves as a valuable foundation for the development of 2D materials f rom MAB phases, holding significant potential for upscaling and expanding the accessibility of other layered 2D materials.

## Supporting Information Available

PXRD patterns and data, SEM images, experimental setup, EDX composition data, HR-TEM images, and XPS survey data (PDF)
