# Supplementary material for: One-Step Synthesis of Metastable Mo2AlB2 from MoAlB Using Gaseous HCl
Source: Inorg Chem. 2025 Jan 6;64(2):1139–45. doi: 10.1021/acs.inorgchem.4c04794 (PMC11752491; doi:10.1021/acs.inorgchem.4c04794)
Supplement: Supplementary file 1 — ic4c04794_si_001.pdf [file ic4c04794_si_001.pdf]

Supporting Information

One-Step Synthesis of Metastable Mo_2AlB_2 from MoAlB Using Gaseous HCl

Tugser Yilmaz [a,b], Ozden Gunes Yildiz [a,b], Naeimeh Sadat Peighambardoust [b], Michael Baitinger [c], Umut Aydemir *[b,d]

[a] *Graduate School of Sciences and Engineering, Koç University, Istanbul-34450, Türkiye*

[b] *Koç University Boron and Advanced Materials Application and Research Center, Istanbul-34450, Türkiye*

[c] *Max-Planck Institute for Chemical Physics of Solids, Nöthnitzer Strasse 40, 01187 Dresden, Germany*

[d] *Department of Chemistry, Koç University, Sariyer, Istanbul, 34450, Türkiye*

* Corresponding author, Email: uaydemir@ku.edu.tr

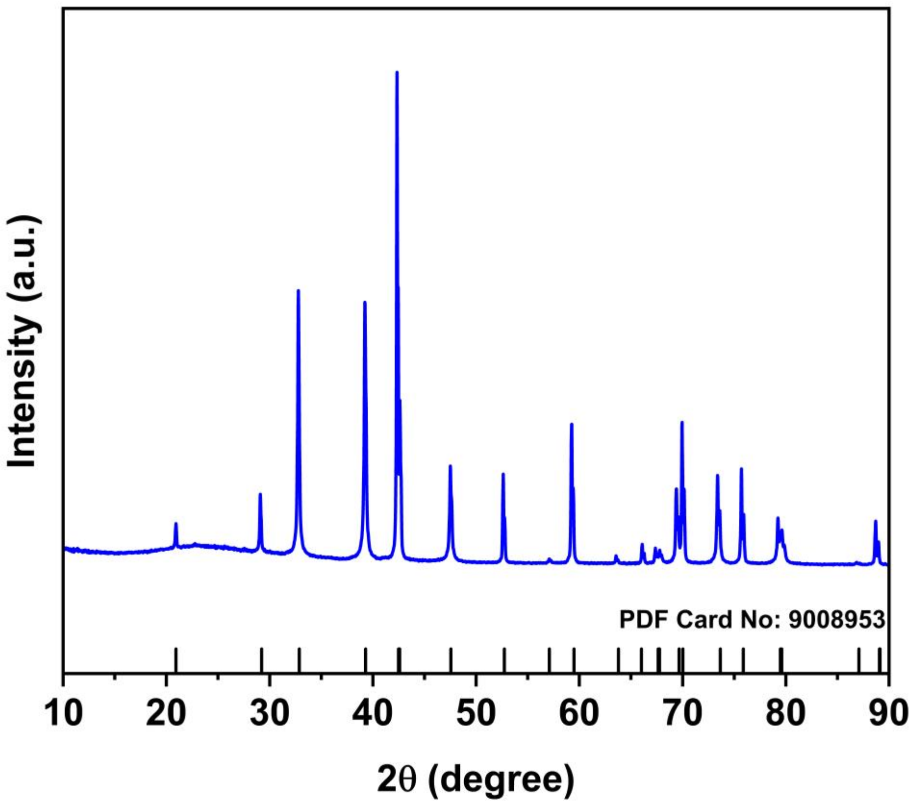

Figure S1. PXRD Pattern of α-MoB obtained from carbothermal reduction.

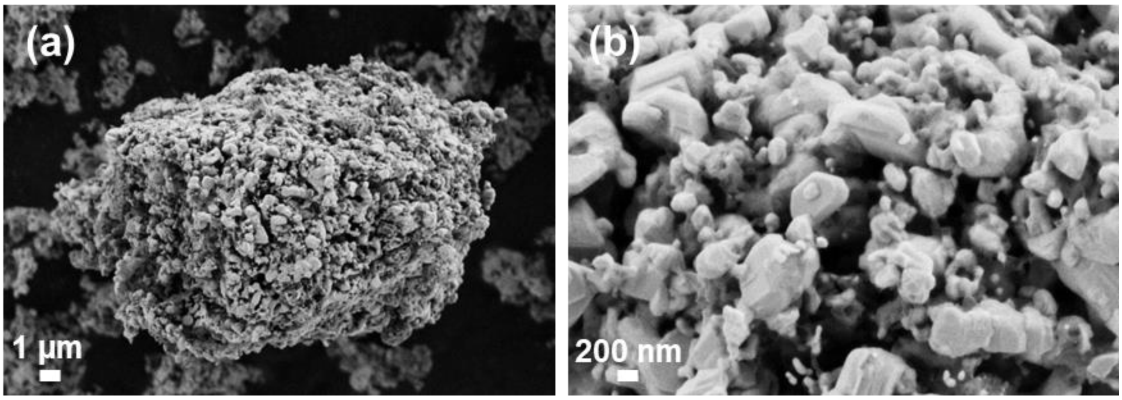

Figure S2. SEM images of the α-MoB with (a) low and (b) high magnification (SE contrast).

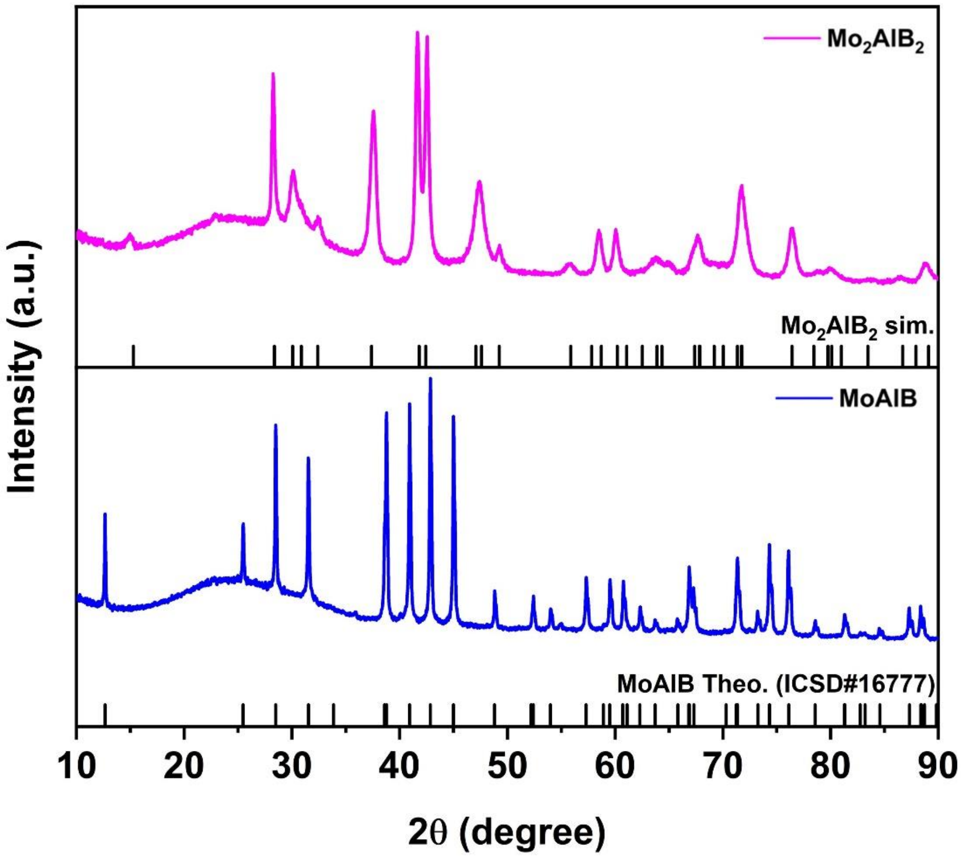

Figure S3. Comparison of the precursor phase MoAlB obtained from solid state reaction (blue pattern) and Mo_2AlB_2 obtained after 2h reaction with gaseous HCl at 450°C (pink pattern). The low-temperature reaction leads to a lower crystallinity and broader reflections.

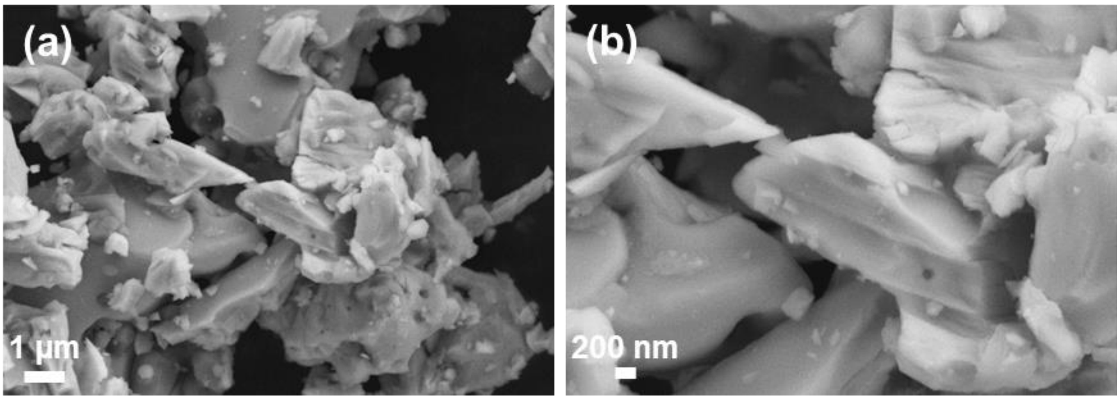

Figure S4. SEM images of MoAlB with (a) low and (b) high magnification (SE contrast).

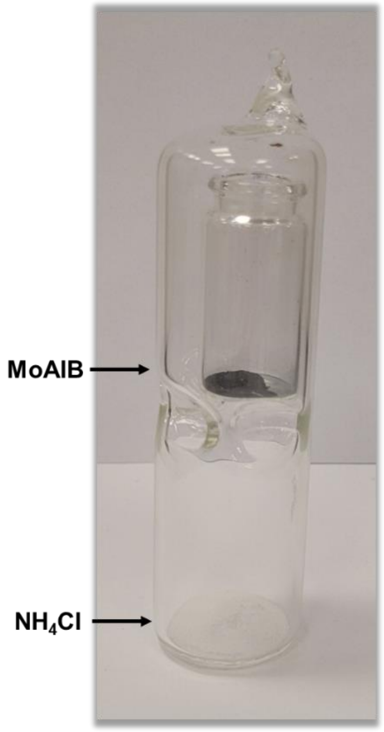

Figure S5. The experimental setup used for the production of Mo_2AlB_2 from MoAlB by gaseous HCl.

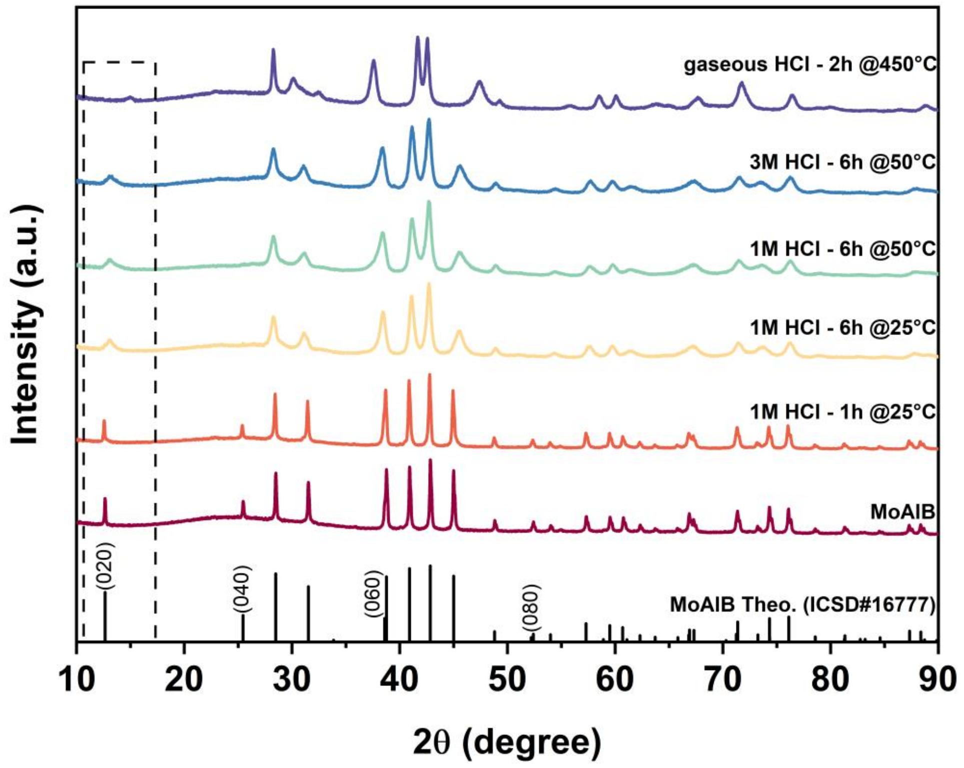

Figure S6. PXRD patterns of MoAlB samples treated with HCl under various conditions, with the gaseous HCl experiment displayed at the top. The hatched area highlights peak positions corresponding to the (020) plane.

Table S1. Atomic percentages of $MoAl_{1-x}B$ samples treated with 1M HCl for 6 hours at 25°C, as determined by SEM-EDX analysis.

Element	at.%
Molybdenum	56.33
Aluminum	36.39
Boron	7.28
**Total**	**100.00**

Table S2. Miller indices, experimental and calculated 2-theta values, and their differences (Delta) used for the calculation of lattice parameters.

h k l	2-theta(e)	2-theta(c)	Delta
0 2 0	14.99	15.311	-0.321
0 0 1	28.229	28.342	-0.113
1 1 0	30.065	30.023	0.042
0 4 0	30.799	30.904	-0.105
0 2 1	32.474	32.367	0.107
1 3 0	37.527	37.36	0.167
1 1 1	41.631	41.758	-0.127
0 4 1	42.503	42.425	0.078
0 6 0	47.067	47.113	-0.046
1 3 1	47.582	47.548	0.034
1 5 0	49.167	49.242	-0.075
0 6 1	55.653	55.896	-0.243
0 0 2	58.461	58.633	-0.172
2 0 0	60.01	60.079	-0.069
1 7 0	63.634	63.902	-0.268
0 8 0	64.787	64.399	0.388
1 1 2	67.513	67.272	0.241
2 0 1	67.961	67.732	0.229
1 7 1	71.568	71.335	0.233
1 3 2	71.751	71.617	0.134
2 4 1	76.337	76.292	0.045
1 5 2	79.919	80.015	-0.096
0 10 0	83.582	83.531	0.051
2 6 1	86.402	86.589	-0.187
2 0 2	88.762	88.892	-0.13

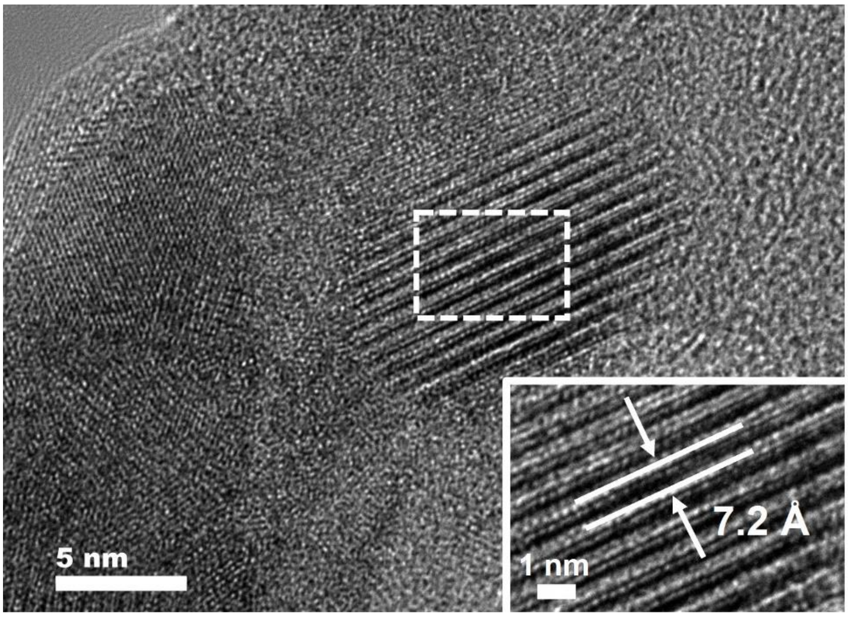

Figure S7. HR-TEM images of MoAlB which corresponds to (020) plane. The interlayer spacing is 7.2Å.

Table S3. Atomic percentage of Mo_2AlB_2 from SEM-EDX.

Element	at.%
Molybdenum	70.87
Aluminum	28.50
Boron	0.63
**Total**	**100.00**

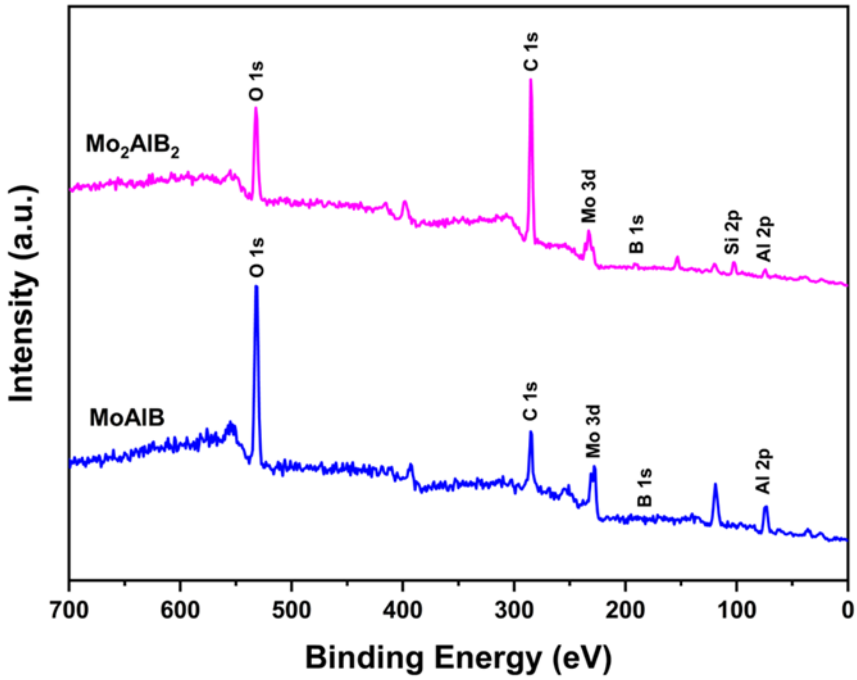

Figure S8. XPS survey of MoAlB and Mo_2AlB_2.
